# Hydrogen Dioxide; a Resume

**Published:** 1891-05

**Authors:** John Aulde

**Affiliations:** Philadelphia, Member of the American Medical Society of the State of Pennsylvania; of the Philadelphia County Medical Society, etc.


### HYDROGEN DIOXIDE; A RESUME.

BY JOHN AULDE, M. D., PHILADELPHIA,
Member of the American Medical Society of the State of Pennsylvania; of the Philadelphia County Medical Society, etc.

Within the past ten years the use of hydrogen dioxide (peroxide of hydrogen) has become quite general among practitioners whose business has led them to give special attention to some particular class of disorders. Many general practitioners, however, have not availed themselves of the benefits afforded by this comparatively recent addition to our therapeutic resources, owing to the expense and the care required in looking after details, together with the uncertainty which attends its employment. These difficulties no longer exist.

Having, then, at our command a remedy possessing such remarkable properties as a bactericide, one which is perfectly harmless when brought into contact with healthy tissues, it will be worth while to study the indications for its use in the treatment of disease.

*Commercial peroxide*, which is used extensively for bleaching purposes and in the arts, is doubtless responsible for unsatisfactory results, but, as compared with the medicinal preparation, it is a very inferior product, sold at a cost of about eight cents a pound. Physicians should know that this product always contains a large proportion of acids (two to five per cent.), hydrofluoric, sulphuric, hydrochloric, oxalic and nitric acids, and, knowing this to be the case, they should be careful to examine the reactions and see that the medicinal preparation obtained by patients is supplied in original packages. The commercial product is not "just as good" nor will it "do as well" for the patient; and if these suggestions are kept in view, the success of the peroxide is assured.

Another important thing which I have learned is, that the mixture of the peroxide with glycerine does not make "glycozone," but, instead, a mixture which generates slowly but constantly secondary products, which appear to possess irritating properties almost as toxic as those of formic acid, well known in Central Africa as a deadly arrow-poison. I am of the opinion also that when the peroxide is used in the form of an inhalation by heating with water, a considerable proportion of the nascent oxygen is transformed into ordinary oxygen before reaching the affected tissues, and while I can readily understand how this must detract from its efficiency, remarkably prompt results have attended its administration in this manner. The only obstacle in the way of securing immediate and favorable results from the exhibition of this agent is our inability to command at all times a freshly prepared and thoroughly reliable product, free from the impurities incident to its manufacture; but that difficulty, I believe, is no longer an excuse, as it can be supplied by the principal druggists throughout the country.

In the light of the foregoing demonstration there can be no hesitancy in ascribing the therapeutical value of oxygen, in whatever form employed, to its influence upon cell activity. The entire organism being composed of cells, the conclusion is inevitable that all agents which increase the normal function of the cell, increase in like manner the resistance of the organism to the inroads of disease. This is further exemplified by the active oxidation (combustion) which takes place when the peroxide is brought into contact with unhealthy tissues, and still no deleterious action is noticeable upon the normal structures, a statement of fact which can be applied to no other known antiseptic. Pus and all other unhealthy discharges are promptly destroyed, the affected structures being left clean and perfectly free from micro-organisms.

*Therapeutics.*—From the peroxide of hydrogen we may obtain in the form of a vapor or spray, the therapeutic effects of nascent oxygen, and as a surgical application or antibacterial substance this product is far superior to the gas itself. Used in the form of a vapor by inhalation, it increases the secondary assimilation by favoring the elimination of excrementitious products through the stimulating effect upon internal respiration. Just as pure mountain air arouses the activity of functions which have been depressed and promotes health, so oxygen evolved in this manner increases tissue change and prevents the suboxidation which attends upon the arrest of cell function. Oxygen is a tissue-builder as well as an oxidizer of carbonaceous and excrementitious products. When it is introduced into the alimentary tract, abdominal fermentations are arrested by the destruction of the germs which produce them; unhealthy mucous secretions are destroyed, while the vitality of the cells lining the walls of the intestine is augmented, and their power against the absorption of ptomaines and leucomaines is greatly increased. The surgeon will find the peroxide an efficient and most convenient antiseptic, as it can be freely used in cavities, in discharging sinuses, and upon the most delicate tissues, without danger of producing the slightest irritation. In all cases of threatened collapse, in low conditions of the system, and during convalescence from severe illness, the physician should bear in mind the wonderful revitalizing properties of this remedy. Perhaps the reader will gain a more practical idea of the applications by a reference to some of the more prominent indications, and I shall briefly pass in review some of the diseases in which it may be used with beneficial results.

In *anœmia and chlorosis, erysipelas, septicœmia. lithœmia, rheumatism.*

It is also of decided benefit in the treatment of *diabetes mellitus* and in *albuminuria*, when it may be presumed to have some active influence in eliminating morbid products.

*Pulmonary affections* have long claimed the attention of those who dabbled with oxygen inhalations, and it is in this class of cases where faithful attention to details will produce most marked effects.

In *surgical practice*, when the solution of the proper strength is brought into contact with diseased tissues, a brisk effervescence takes place and continues until all the pus-corpuscles present are destroyed. This solution may be used topically in nearly all cases of catarrh of the upper air passages in the form of spray, and it may be used as an antiseptic after the removal of pus in *empyema*. The substance possesses the advantage over other antiseptics of being harmless, and can therefore be used freely in *diphtheria* and *croup*. There are so many indications for its employment that it would be difficult to mention all the *topical uses*, although the following may be referred to, viz.: boils, carbuncles, indolent ulcers, carcinoma, and venereal diseases as an injection.

The most flattering commendations of "Marchand's peroxide of hydrogen (medicinal)" have been given voluntarily by numerous well known authors and contributors to medical literature within the past few years. Dr. Morris refers to it as "the necessary peroxide of hydrogen," and I have found Marchand's product to possess in a remarkable degree the properties so essential to success—viz.: uniformity in strength, purity and stability.—*New York Medical Journal.*

